# Resistance Evolution Against Antimicrobial Peptides in *Staphylococcus aureus* Alters Pharmacodynamics Beyond the MIC

**DOI:** 10.3389/fmicb.2020.00103

**Published:** 2020-02-14

**Authors:** Baydaa El Shazely, Guozhi Yu, Paul R. Johnston, Jens Rolff

**Affiliations:** ^1^Evolutionary Biology, Institute for Biology, Free University of Berlin, Berlin, Germany; ^2^Zoology Department, Faculty of Science, Alexandria University, Alexandria, Egypt; ^3^College of Life Sciences, Sichuan Agricultural University, Ya’an, China; ^4^Berlin Center for Genomics in Biodiversity Research, Berlin, Germany; ^5^Leibniz-Institute of Freshwater Ecology and Inland Fisheries (IGB), Berlin, Germany; ^6^Berlin-Brandenburg Institute of Advanced Biodiversity Research, Berlin, Germany

**Keywords:** resistance evolution, Hill coefficent, pharmacodynamics, pexiganan, melittin

## Abstract

Antimicrobial peptides (AMPs) have been proposed as a promising class of new antimicrobials partly because they are less susceptible to bacterial resistance evolution. This is possibly caused by their mode of action but also by their pharmacodynamic characteristics, which differ significantly from conventional antibiotics. Although pharmacodynamics of antibiotic resistant strains have been studied, such data are lacking for AMP resistant strains. Here, we investigated if the pharmacodynamics of the Gram-positive human pathogen *Staphylococcous aureus* evolve under antimicrobial peptide selection. Interestingly, the Hill coefficient (kappa κ) evolves together with the minimum inhibition concentration (MIC). Except for one genotype, strains harboring mutations in *men*F and *atl*, all mutants had higher kappa than the non-selected sensitive controls. Higher κ results in steeper pharmacodynamic curve and, importantly, in a narrower mutant selection window. *S. aureus* selected for resistance to melittin displayed cross resistant against pexiganan and had as steep pharmacodynamic curves (high κ) as pexiganan-selected lines. By contrast, the pexiganan-sensitive tenecin-selected lines displayed lower κ. Taken together, our data demonstrate that pharmacodynamic parameters are not fixed traits of particular drug/strain interactions but actually evolve under drug treatment. The contribution of factors such as κ and the maximum and minimum growth rates on the dynamics and probability of resistance evolution are open questions that require urgent attention.

## Introduction

Bacterial drug resistance is a growing problem (Davies and Davies, 2010). Under conventional antibiotic treatment resistance evolves frequently (Levy and Marshall, 2004; Davies and Davies, 2010). Solving this problem requires new approaches including prudent use, understanding evolutionary dynamics (zur Wiesch et al., 2014) and the identification of new drug candidates (WHO, 2012) that are likely to avoid evolution of resistance (Czaplewski et al., 2016). Antimicrobial peptides (AMPs) have been proposed as promising new drug candidates (Zasloff, 2002; Fjell et al., 2012; Mylonakis et al., 2016; Pfalzgraff et al., 2018). Though resistance against AMPs evolves readily in *in vitro* systems (Perron et al., 2006; Habets et al., 2012; Dobson et al., 2013; Makarova et al., 2018), this does not seem to be the case *in vivo*. Based on pharmacodynamic studies of AMPs, one of their alleged advantage is that evolution of resistance has a much lower probability compared to antibiotics (Yu et al., 2018).

Pharmacodynamics are based on time-kill curves. Regoes et al. (2004) analyzed time-kill curves using a pharmacodynamic model that is closely related to Emax models (Mueller et al., 2004). Pharmacodynamic functions link drug dosage and bacterial growth or death rates. Four parameters are important for this model (Regoes et al., 2004): the Hill coefficient (κ), i.e., the slope, the maximal bacterial growth rate in the absence of antimicrobial (Ψ_max_), the minimal bacterial growth rate at high concentrations of antimicrobial (Ψ_min_), and the pharmacodynamic minimum inhibition concentration (zMIC) (Regoes et al., 2004).

The steepness of pharmacodynamic curves, as described by κ, is much greater for AMPs than for antibiotics (Yu et al., 2016). The maximum killing effect of AMPs is much stronger than that of antibiotics, as measured via the speed of killing (Fantner et al., 2010; Yu et al., 2018). Consequently, AMPs display a narrower mutation selection window compared to antibiotics, thus resistance toward AMPs is less likely to evolve (Yu et al., 2018). Moreover, AMPs cocktail have higher kappa values than single AMPs (Yu et al., 2016); a crucial information for combinational therapy, a proposed antibiotic replacement regimes (Walkenhorst, 2016).

Despite short comings, the minimum inhibition concentration (MIC) is still the most common bioassay to explore cross resistance (Brauner et al., 2016; Wen et al., 2016). The importance of pharmacodynamic parameters in predicting drug resistance evolution has been reported in several studies (Chevereau et al., 2015; Lukačišinová and Bollenbach, 2017). In addition to *in vivo* infection dynamics studies (Dobson et al., 2014; McGonigle et al., 2016; Zanchi et al., 2017; El Shazely et al., 2019), pharmacodynamic approaches are useful to understand how antibiotics and antimicrobial peptides eradicate bacteria in physiological systems (Yu et al., 2016). It is assumed that the shape of pharmacodynamic curve does not change (Craig, 1998).

Here, we use a pharmacodynamic approach, that has been previously described to generate a sigmoid dose–response relationship (Bonapace et al., 2002; Regoes et al., 2004; Sampah et al., 2011; Yu et al., 2016), to study the evolution of AMP resistance. We explored whether the steepness of the pharmacodynamic curve (described by the Hill coefficient, κ), can evolve. It is the first time that pharmacodynamic parameters (kappa, Ψ_min_, Ψ_max_) of AMP resistant strains have been investigated. In this study, a standardized *in vitro* time-kill curve assay for the human pathogen, *Staphylococcous aureus*, which has been selected against either pexiganan, melittin (this study), tenecin 1 or tenecin 1 + 2 (strains from our previous study (Makarova et al., 2018)) was performed. We address two questions. (i) Do kappa, Ψ_min_, Ψ_max_ evolve? (ii) Does cross resistance or cross sensitivity influence the pharmacodynamic parameters: kappa, Ψ_min_, Ψ_max_ and zMIC?

## Materials and Methods

### Bacterial Strains and Culturing Conditions

*Staphylococcus aureus* SH1000 was used in this study. Non-cation adjusted Mueller Hinton Broth (MHB) (PanReac AppliChem, Cat #413788-1210) was used for bacterial cultures. Bacterial cultures were incubated at 37°C with shaking at 180 rpm and plated on Mueller Hinton Agar (MHA). The later was prepared by adding 15 g/l bacteriological Agar Agar (Carl Roth, Cat #2266.2) to MHB.

### Antimicrobial Peptides

We used two different AMPs: pexiganan and melittin. Pexiganan is a synthetic AMP, an analog of Magainin II that was originally isolated from the epidermis of the African clawed frog, *Xenopus laevis* (Zasloff, 1987) (Pexiganan was a kind gift from Michael Zasloff). Melittin (purity > 95%, GenScript, Cat #RP10290) is a synthetic AMP known to be an analog of the main toxin of bee venom (Habermann, 1972). To avoid multiple freeze-thaw cycles, peptides were re-suspended in (1:1 v/v) sterile distilled water and glycerol (Sigma life science, Cat #G5516) to the final concentration of 5 mg/ml and stored at −20°C in sterile vials.

### Selection Experiment

The selection experiment was done according to Makarova et al. (2018). Briefly, preadapted *S. aureus* SH1000 glycerol stocks were stored at −80°C from the above-mentioned study. Five preadapted strains were inoculated into 10 ml MHB and incubated overnight with shaking at 37°C. The cultures were then diluted 1:1000 and incubated at 37°C in 50 ml polypropylene Falcon tubes (Th.Geyer, Cat #7696724) containing 3.7 ml MHB. Short pre-adaptation was carried out by serial passage every 24 h for 4 days, with daily measurements of optical density at 600 nm, contamination checks by plating out on MHA and cryopreservation of culture aliquots at −80°C in 12% glycerol solution.

For the selection protocol, the experiment was performed at 37°C in a microplate reader (Synergy 2, Biotek). To avoid attachment of the peptides to the plastic surfaces, flat bottom polypropylene non-binding 96-well plates were used (Greiner Bio-One, Cat #655261). To minimize evaporation, the 96-well plates were covered with clear polystyrene lids with condensation rings (Greiner Bio-One, Cat #656171). The plates were filled with 200 μl of MHB per well. Growth dynamics were monitored by optical density measurement at a wavelength of 600 nm every 10 min. Measurements were preceded by a moderate shaking for 10 s and continued for 24 h. For each of the five replicate lines there were two experimental conditions: pexiganan or melittin, as well as two controls: negative control, and non-selected control.

The serial passage started at 1 μg/ml for pexiganan (MIC of the preadapted strains toward pexiganan was 2–4 μg/ml) and 2 μg/ml melittin (MIC of the preadapted strains toward melittin was 4–8 μg/ml). Overnight cultures of the five replicate lines were diluted 1:100 and sub-cultured until OD_600_ of 0.5. Ten μl of these cultures were inoculated into each treatment and control wells resulting in final total volume of 200 μl. Four μl (2%) of the culture were transferred every 24 h to a fresh 96 well plate. The concentration of AMPs was doubled each week. Plates were regularly checked for contamination. Glycerol was added to the rest of the cultures to the final concentration of 12% and the plates were stored at −80°C. During the selection experiment, a strain required 5–7 days to evolve resistance such that the culture could survive a two-fold increase in the AMP concentration. The selection experiment continued for 8 weeks where MIC was duplicated 64 folds for both pexiganan and melittin (64×*MIC*).

### Antimicrobial Susceptibility Testing

Minimal inhibitory concentration (MIC) was determined using a broth micro-dilution method. Briefly, 5 μl (1 × 10^5^ CFU/ml) of 1:100 dilution of the mid-exponential phase bacterial culture (OD_600_ = 0.5) were inoculated into the wells of polypropylene V-bottom 96-well plates (Greiner Bio-One,Cat #651261) containing two-fold dilution series of the stressor in a total volume of 100 μl MHB per well. The assay was performed in triplicate. The plates were incubated at 37°C in a humidity chamber. The lowest concentration that inhibited visible bacterial growth after 24 h of incubation is the MIC. Visual observations were confirmed by heat maps generated by Gen 5 software (Biotik) of OD_600_ measurements on a microtiter plate reader (Synergy 2, Biotek).

### Growth Curves

Growth curve assays were performed using a microtiter plate reader (Synergy 2, Biotek). The changes in turbidity at OD_600_ of the selected mutant lines and the non-selected controls were monitored in un-supplemented MHB. For this, 20 μl of 1:10 dilution of mid exponential phase of bacterial culture were inoculated into 180 μl MHB. Each assay had three replicates. The measurements were taken at 10 min intervals during 38 h of incubation at 37°C, with 5 s shaking before each reading. Growth parameters such as final OD, the maximum growth rate (Vmax) and lag time were calculated with Gen5 software (Biotek).

### DNA Isolation

Genomic DNA for whole genome sequencing was isolated using Quick-DNA Fungal/Bacterial Microprep kit (Zymo Research, Cat #D6007) following manufacturer’s instructions. Briefly, 2 × 10^8^ log phase bacteria were resuspended in 200 μL of phosphate buffer saline (PBS) (pH = 7.4, Chem solute, Cat #8035.0100) solution. Then, 750 μL of bashing beads buffer were added and the mixture was transferred into bashing beads lysis tubes. The tubes were placed in a homogenizer (Retsch MM 400) at maximum speed for 5 min. The mixture was centrifuged shortly at 10,000 g for 1 min. Then, 400 μL of supernatant were transferred to a spin filter. The filtrate was chemically lysed by adding 1200 μL genomic lysis buffer. Then the mixture was passed into a zymo-spin IC column, centrifuged and washed twice, first, with DNA Pre-wash buffer, then, with DNA wash buffer. Finally, the DNA was eluted using 20 μL of DNA elution buffer. The DNA quantity and quality were estimated by measuring the optical density at A260/280 using the Nanodrop spectrophotometer (Thermo Scientific) and agarose gel electrophoresis.

### Sequencing

To identify mutations in experimentally evolved populations and strains, the haploid variant calling pipeline snippy v3.2 (Seemann, 2015) was used with default parameters [minimum read mapping quality (–mapqual) 60, minimum base quality (–basequal) 20, minimum coverage (–mincov) 10, and minimum proportion of variant coverage (minfrac) 0.9] as previously described in Makarova et al. (2018). Briefly, quality-filtered adaptor-trimmed reads were aligned to the SH1000 reference genome using bwa (Li, 2013). The Bayesian genetic variant detector freebayes (Garrison and Marth, 2012) was used to detect single-nucleotide polymorphisms, insertions, deletions, multi-nucleotide polymorphisms, as well as composite insertion and substitution events.

### Killing Curves

For pexiganan- and melittin-selected strains, pexiganan was serially diluted (two-fold concentration gradient), starting with 256 × MIC (1024 μg), in 96-well plate. Approximately, 2–3 × 10^6^ log-phase bacteria were added to a total volume of 100 μl. The plates were incubated at 37°C. Killing by AMPs is rapid (Sochacki et al., 2011; Rangarajan et al., 2013), therefore dose response was monitored within 60 min (Yu et al., 2016). Ten microliters of bacterial suspension were taken out after 30 s and then every 20 min, then immediately diluted in PBS and plated on square solid MHA plates. These solid agar plates were transferred into 30°C incubator. CFU were counted 24 h later. The incubation of plates at 30°C facilitate counting CFU before colonies overgrow and overlap. The limit of detection in our system is 14 CFUs.

Following the same protocol, we determined the killing curves for the tenecin 1 and tenecin 1 + 2-selected strains available in the laboratory from a former study (Makarova et al., 2018). For these 36 genotypically unique strains, pexiganan was serially diluted starting with 16 × MIC (64 μg), to save material as it was known from MIC results that they do not share cross resistance to pexiganan.

### Data Analysis

Statistical analysis was done in R (R Core Team, 2013).

#### MIC Analysis

The best fit was obtained when the MIC values were log_2_ transformed. A linear model was fitted to the transformed data. Treatment and mutation were considered as explanatory variables in the model. Several normality checking functions were used to test normality assumptions such as “bptest” (Breusch-Pagan test against heteroskedasticity) and “dwtest” (Durbin-Watson test for autocorrelation of disturbances) from “lmtest” package (Hothorn et al., 2019), “gvlma” (Global Validation of Linear Models Assumptions) from “gvlma” package (Pena and Slate, 2012) and “durbinWatsonTest” from “car” package (Fox et al., 2012). The function “anova” was used to analyze the model and extract *F*-statistics and degrees of freedom. The “mean”, “sd” and “var” functions were used to calculate the mean, standard deviation, standard error and variance. For analysis of contrasts, *post hoc* comparisons were performed using “lsmeans” function with a “tukey” adjustment (Lenth and Lenth, 2018). We used the function “visreg” from package “visreg” (Breheny et al., 2019) to visualize the contrast plot of the treatment effect as extracted from the model.

#### Growth Curve Analysis

Growth parameters (Vmax, duration of lag phase and final OD_600_) were analyzed by using the “lm” function for linear models. For contrasts, *post hoc* tests were performed using “lsmeans” and “visreg” functions as described before.

#### Modeling Killing Curves

A Hill function was used to model the killing curve as previously described (Regoes et al., 2004; Yu et al., 2016). Briefly, generalized linear regression was used to determine growth and killing rates of bacteria from the time-kill curves as the change of CFUs over time. The CFU data were log-transformed (log_10_). Using the rjags package (Plummer et al., 2016), the growth and killing rates were fitted and extracted based on Markov Chain Monte Carlo (MCMC) method and the pharmacodynamic curves were generated.

#### Analysis of Pharmacodynamic Parameters

The pharmacodynamic parameters were extracted from the MCMC output. We tested whether the pharmacodynamic MIC of various *S. aureus* strains selected against AMPs segregated by selection treatment and/or by mutation. The zMIC was log_2_ transformed and a linear model was fitted as described above. A generalized linear model with gamma distribution was fitted to analyze Ψ_max_ variances across *S. aureus* strains with different selection treatment and different mutation. The Ψ_min_ variances were analyzed using a linear model. The Hill coefficient κ data set was normalized by log-transformation (log_10_) then a linear model was fitted. *Post hoc* analysis was performed as described above.

## Results

### Resistance Evolved at a Cost

**FIGURE 1:**
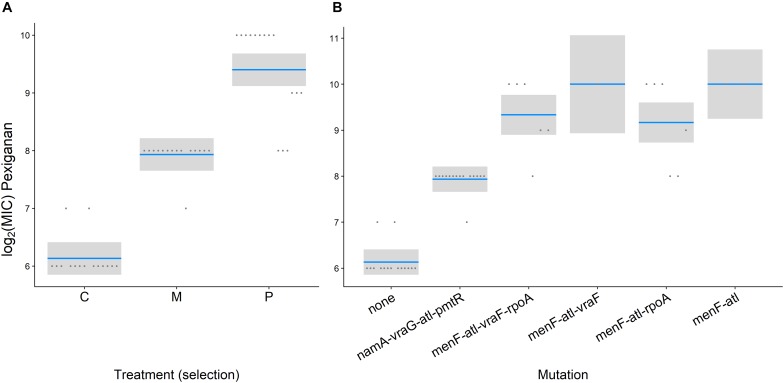
Log_2_ MIC of pexiganan and melittin resistant *S. aureus* strains compared to procedural controls tested against pexiganan separated by treatment **(A)** or by mutation **(B)** (C and none: unselected control, M: melittin-selected strains with mutation in *nam*A, *vra*G, *atl*, and *pmt*R gene loci, P: pexiganan-selected strains segregated into four genotype variants with mutation in *men*F, *atl*, and *vra*F, *rpo*A or both, blue line: mean, gray box: 95% confidence intervals). According to minimum inhibition sensitivity test, MIC _pexiganan_ segregated by treatment (*F*_(__2_,_40__)_ = 143.2300, *p* < 0.0001, Figure 1A) but not by mutation (*F*_(__2_,_40__)_ = 1.8769, *p* = 0.166, Figure 1B).

After 8 weeks of selection, all lines were able to grow in the presence of 256 μg/ml pexiganan or 512 μg/ml melittin, which is equivalent to 64-fold of MIC of non-selected preadapted strains for both stressors. According to minimum inhibition sensitivity test, MIC _pexiganan_ segregated by treatment (*F*_(__2_,_40__)_ = 143.2300, *p* < 0.0001, Figure 1A), but not by mutation (*F*_(__2_,_40__)_ = 1.8769, *p* = 0.166, Figure 1B). *S. aureus* evolved pexiganan resistance both when selected against pexiganan (*T* = 16.554, df = 42, *p* < 0.0001, Figure 1) and melittin (*T* = 9.121, df = 42, *p* < 0.0001, Figure 1). Moreover, MIC _pexiganan_ differed significantly between pexiganan- and melittin-selected lines (*T* = 7.432, df = 42, *p* < 0.0001, Figure 1A). Pexiganan-selected lines showed cross resistance to melittin (*T* = 8.457, df = 42, *p* < 0.0001, Supplementary Table S1).

**FIGURE 2:**
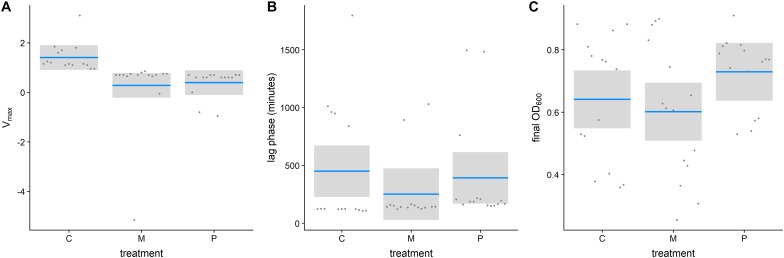
Growth parameters of evolved AMP-resistant *Staphylococcus aureus* in relation to treatment [Vmax **(A)**, lag phase **(B)**, final OD **(C)**]. (Con: unselected control, M: melittin-selected strains, P: pexiganan-selected strains, blue line: mean, gray box: 95% confidence intervals).

Antimicrobial peptides-selected strains had consistently slower growth rates in the exponential phase for both, pexiganan (Vmax: *T* = 2.821, df = 42, *p* = 0.01, Figure 2A) and melittin-selected strains (Vmax: *T* = 3.146, df = 42, *p* = 0.008, Figure 2A) compared to non-selected controls (Supplementary Figure S1). However, lag phases (lag _Pexiganan_: *T* = 0.356, df = 42, *p* = 0.932, lag _Melittin_: *T* = 1.234, df = 42, *p* = 0.440, Figure 2B) and final population sizes measured as OD did not differ (Final OD_Pexiganan_: *T* = –1.313, df = 42, *p* = 0.396, Final OD_Melittin_: *T* = 0.592, df = 42, *p* = 0.825, Figure 2C).

### Genome Sequencing Reveals Mutations in a Number of Loci Related to Selection Treatment

Whole genome sequencing of the selected mutants and the non-selected controls (at the single colony level) showed differences between treatments. In each melittin-resistant strain at least four mutations in *pmt*R (also known as *ytr*A), *vra*G (also known as *bce*B), *atl*, and *nam*A genes were identified. All those mutations are known to be involved in cell wall stress tolerance and detoxification (see also Supplementary Table S2 for a full list of mutations). The mutations included stop gain for *pmt*R, missense (c.1727C > T p.Ala576Val for 8 strains and c.924T > A p.Ser308Arg for nine strains) for *vra*G, frameshift for *atl* (c.2705_2706dupAT p.Ala903fs) and synonymous (c.477G > A p.Ala159Ala) for *nam*A. Interestingly, we found the same *pmt*R stop-gain mutation (c.77T > A p.Leu26^∗^) in all melittin-selected strains in this study, which has previously been described for melittin-selected *S. aureus* lines (Johnston et al., 2016) and for tenecin-selected *S. aureus* (Makarova et al., 2018). For all pexiganan-selected strains, a missense mutation (c.571C > G p.Arg191Gly) for *men*F gene and stop gains and disruptive in-frame deletion mutations (c.2331_2342delTACTGTTACTAA p.Tyr777_Lys781delinsTer) for *atl* gene were consistent. Two strains only harbored these two mutations, while the other pexiganan-selected strains carried additional mutations either conservative in-frame insertion (c.178_192dupTCACAAGGTTCTATT p.Ser60_Ile64dup) in *vra*F gene (also known as *bce*A), or missense (c.884C > T p.Ser295Phe) in *rpo*A gene or both. Six pexiganan mutants had a missense in *rpo*A, one had conservative in-frame insertion in *bce*A gene while six strains had mutations in both loci.

### Killing (Dose-Response) and Pharmacodynamic Curves

We tested *in vitro* killing of pexiganan using different AMP resistant *S. aureus* strains (pexiganan, melittin, tenecin 1 and tenecin 1 + 2-selected strains) and their respective controls. Time-kill curves were obtained by counting viable CFUs after treatment with various concentrations of pexiganan (Supplementary Figure S2). The CFUs of surviving bacteria strongly decreased as a function of time at higher pexiganan concentrations, however slight increases were noticed at lower concentrations. There are four measurements of the bacterial density during the first 60 min after exposure to pexiganan. This time interval was appropriate to statistically estimate the bacterial net growth rate at a given concentration and construct the pharmacodynamic curves (Supplementary Figure S3).

The average values of pharmacodynamic MICs (zMIC) and those determined by a two-fold dilution protocol (MIC) are listed in Table 1. The estimated pharmacodynamic zMICs differs slightly from the MIC measurements.

**TABLE 1:** Parameter estimates and their standard errors.

**S. aureus**	**MIC (μg/ml)**	**zMIC (μg/ml)**	**Ψ_min_ (h**^–^**^1^)**	**Ψ_max_ (h**^–^**^1^)**	**κ**
Con1	72.535.81	66.767.42	−9.900.22	0.230.06	2.860.13
P	768.0086.54	300.3223.33	−7.600.30	0.260.06	**15.32 ± 1.32**
M	247.468.53	283.1930.16	−8.200.21	0.110.03	**15.10 ± 1.42**
Con2	48.007.16	22.943.77	−9.040.16	0.460.07	4.840.53
T1	49.783.86	21.022.06	−8.860.13	0.410.03	4.020.34
T1T2	53.334.54	19.292.86	−7.970.35	0.270.03	4.180.69

*MIC, minimum inhibition concentration determined by a two-fold dilution protocol; zMIC, pharmacodynamic MIC; Ψ_*min*_, the minimum bacterial net growth rate at high pexiganan concentrations; Ψ_*max*_, the maximum bacterial net growth rate; κ, the Hill coefficient; Con, passaged non-selected control for pexiganan- and melittin-selected lines (Con1) and for tenecin-selected lines (Con2); P, pexiganan-selected *S. aureus;* M, melittin-selected *S. aureus;*T1, tenecin 1-selected *S. aureus;* T1T2, tenecin1 + 2-selected *S. aureus.* Pexiganan (P) and Melittin (M) selected *S. aureus* lines evolved higher kappa values (Bold) compared to their unselected controls (Con1).*

**FIGURE 3:**
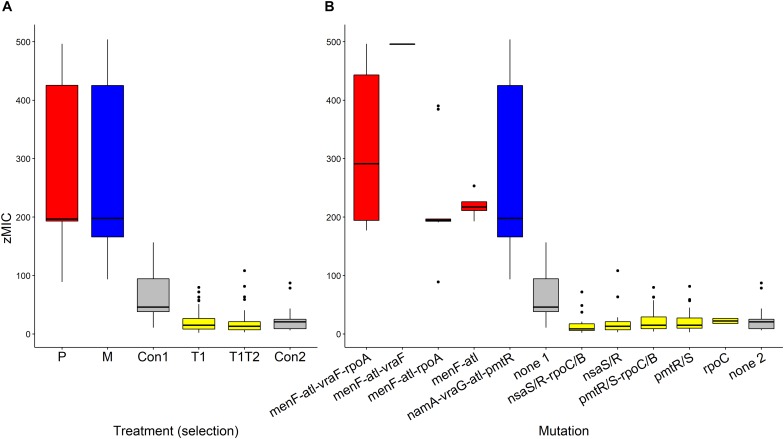
Estimated pharmacodynamic MIC (zMIC) of AMP-selected versus AMP-sensitive *S. aureus* segregated by treatment **(A)** or by mutation **(B)**. (Con: passaged non-selected control for pexiganan- and melittin-selected lines (Con1, none1, gray) and for tenecin-selected lines (Con2, none2, gray), P: pexiganan-selected *S. aureus* (red) segregated into 4 genotype variants with mutation in *men*F, *atl*, and *vra*F, *rpo*A or both, M: melittin-selected *S. aureus* (blue) with mutation in *nam*A, *vra*G, *atl*, and *pmt*R gene loci,T1: tenecin 1-selected *S. aureus* (yellow) and T1T2: tenecin1 + 2-selected *S. aureus* (yellow) harboring a mutation in either *pmt*R/S or *nsa*S/R gene loci which might be accompanied by a mutation in *rpo*C/B). The boxplots show the first to the third quartiles and the median. The bars indicate the 1.5 interquartile of the lower and upper quartiles. The dots represent outliers.

The pharmacodynamic MIC (zMIC) segregated by treatment (*F*_(__5_,_283__)_ = 127.67, *p* < 0.0001, Figure 3A). The pexiganan resistant strains had a zMIC higher than its respective control (*T* = 9.451, df = 238, *p* < 0.0001, Figure 3A). Melittin resistant strains had a cross resistance to pexiganan (*T* = 7.911, df = 238, *p* < 0.0001, Figure 3A). The zMIC values of pexiganan- and melittin-selected *S. aureus* did not differ significantly (*T* = 0.542, df = 238, *p* = 0.9944, Figure 3A). Tenecin 1 and tenecin 1 + 2 strains were as sensitive to pexiganan as their respective non-selected controls (tenecin1-control: *T* = –0.782, df = 238, *p* = 0.9703; tenecin1 + 2 -control: *T* = –1.522, df = 238, *p* = 0.6503, Figure 3A). zMIC did not segregate by mutation within each treatment, for example *vra*F (*bce*A) mutants are not significantly different from *men*F-*atl* mutants (*T* = 1.569, df = 232, *p* = 0.9184, Figure 3B).

### Ψ_max_ and Ψ_min_

**FIGURE 4:**
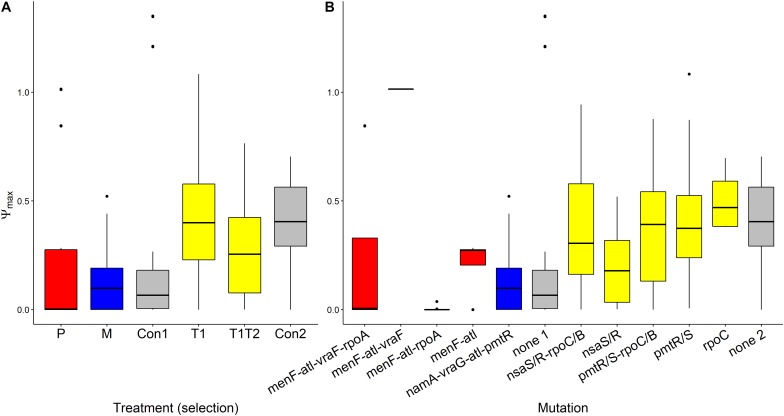
Variation of Ψ_max_ values of AMP-selected versus AMP-sensitive *S. aureus* predicted by MCMC segregated by treatment **(A)** or by mutation **(B)**. Ψ_min_ refers to the maximal growth rate of bacteria at low pexiganan concentration. The Boxplot description, abbreviations and color reference are previously described in Figure 3.

Ψ_max_ values were found to be almost identical across treatments (Figure 4). This suggests that growth rates of bacteria in low concentration of AMP(s) were presumably close to the natural growth rate (Yu et al., 2016). It is interesting that differences in Vmax, referred to earlier, were not reflected by differences in Ψ_max_ values.

**FIGURE 5:**
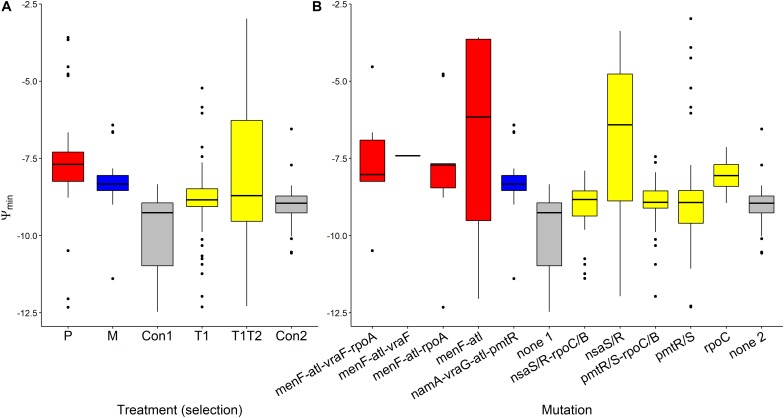
Variation of Ψ_min_ of AMP-selected versus AMP-sensitive *S. aureus* predicted by MCMC segregated by treatment **(A)** or by mutation **(B)**. Ψ_min_ describes minimal growth rate of bacteria under at high pexiganan concentration. Ψ_min_ values segregated by treatment (*F*_(__5_,_232__)_ = 10.285, *p* < 0.0001). The Boxplot description, abbreviations and color reference are described in Figure 3.

Ψ_min_ values segregated by treatment (*F*_(__5_,_232__)_ = 10.285, *p* < 0.0001, Figure 5A). Pexiganan- and melittin-selected *S. aureus* had higher Ψ_min_ values than the non-selected controls (P-Con: *T* = 6.130, df = 238, *p* < 0.0001; M-Con: *T* = 4.049, df = 238, *p* = 0.001, Figure 5). The Ψ_min_ values for tenecin 1- and tenecin 1 + 2-selected strains were not different from non-selected controls (T1-C: *T* = 0.511, df = 238, *p* = 0.995; T1T2-C: *T* = 2.759, df = 238, *p* = 0.0678, Figure 5A). However, tenecin 1-selected *S. aureus* had a slightly lower Ψ_min_ values than tenecin1 + 2-selected strains (*T* = –3.037, df = 238, *p* = 0.0315, Figure 5A). Pexiganan resistant strains had almost equal Ψ_min_. Tenecin-selected *S. aureus* with mutation in the *nsa* operon had uniquely higher Ψ_min_ than the non-selected control (*T* = 5.303, df = 232, *p* < 0.0001, Figure 5B), having an additional mutation in the *rpo* operon at C or B loci would decrease the Ψ_min_ values significantly (*T* = –5.494, df = 232, *p* < 0.0001, Figure 5B).

### Does Kappa Evolve?

**FIGURE 6:**
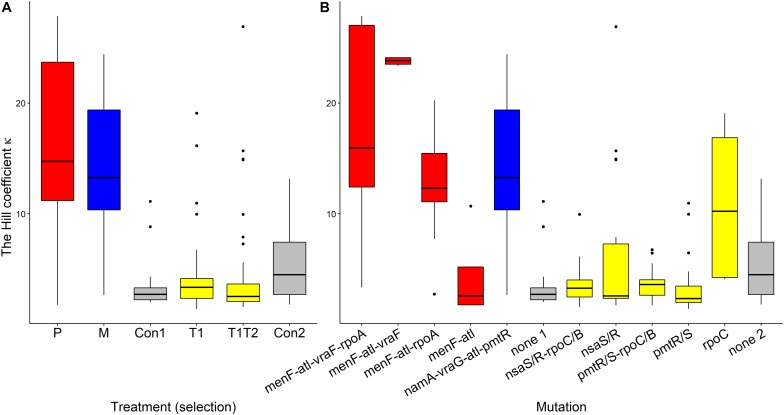
Variations of Hill coefficients κ of AMP-selected versus AMP-sensitive *S. aureus* predicted by MCMC segregated by treatment **(A)** or by mutation **(B)**. Kappa predicts the shape slope of the pharmacodynamic curve, the larger the κ value, the steeper is the pharmacodynamic curve. The Boxplot description, abbreviations and color reference are described in Figure 3.

Pexiganan-selected *S. aureus* had significantly higher kappa values than non-selected controls (*T* = 11.191, df = 238, *p* < 0.0001, Table 1 and Figure 6A), resulting in very steep pharmacodynamic curves (Supplementary Figure S3). Pexiganan-selected strains lacking mutations in both *vra* (*bce)* and *rpo* operons showed however a kappa value as low as the non-selected controls (*T* = 0.570, df = 232, *p* = 1.00, Figure 6B); therefore, a shallower pharmacodynamic curve compared to pexiganan resistant mutants (Supplementary Figure S3). The cross resistance of melittin-selected strains toward pexiganan seemed to result in kappa values greater than in the control strains (*T* = 10.353, df = 238, *p* < 0.0001, Table 1 and Figure 6A). Tenecin-selected strains were as sensitive as their non-selected control and for that kappa was consistent (T1-Con: *T* = –1.521, df = 238, *p* = 0.651; T1T2-Con: *T* = –2.147, df = 238, *p* = 0.267, Table 1 and Figure 6A). However, tenecin-selected strains with mutations in the *pmt* operon had a kappa value lower than non-selected controls (*T* = –3.617, df = 232, *p* = 0.0185, Figure 6B).

## Discussion

Our study was probably the first to explore the co-evolution of pharmacodynamic parameters (κ, Ψ_min_, Ψ_max_) and bacterial AMP resistance. We found that AMP resistance evolution in *S. aureus* resulted not only in increasing MICs, but, importantly in some strains also in changes in the Hill coefficient (κ), resulting in steeper pharmacodynamic curves. Kappa evolved in a mutation dependent manner. Despite the slower AMP selected bacterial growth rate, as captured by Vmax extracted from growth curve analysis, the maximum bacterial growth rate in absence of pexiganan, Ψ_max_, did not evolve. The maximum killing rate at high concentration of pexiganan, Ψ_min_, co-evolved with *in vitro* AMP selection. Cross resistance (melittin-selected strains) and cross sensitivity (tenecin 1- and tenecin 1 + 2-selected strains) affected both zMIC and kappa but not Ψ_min_ and Ψ_max_.

Our selection protocol covered several weeks. This is consistent with treatment regimens for complicated *S. aureus* infections, where 4–6 weeks of intravenous therapy has been the standard practice for over half a century and continues to be recommended (Tong et al., 2015). Although *in vitro* selection of *S. aureus* (SH1000) against tenecin 1 and a combination of tenecin 1 + 2 lasted for 8 days (Makarova et al., 2018), following the same protocol herein, to evolve pexiganan and melittin resistance required 8 weeks. Moreover, in one of our former studies *S. aureus* (JLA513) extinction was observed after 2 weeks of pexiganan selection (Dobson et al., 2013), which was not repeated in the current study. The explanation of differences in pathogen/drug evolutionary dynamics remains poorly understood (Chevereau et al., 2015).

The fitness cost of a pathogen can be inferred from a reduced growth rate *in vivo* (Majcherczyk et al., 2008) or *in vitro* (zur Wiesch et al., 2010). Here, we find clear evidence for costly resistance as measured in slower growth rate (Vmax). However, unlike tenecin-selected lines (Makarova et al., 2018), lag phase for both pexiganan and melittin AMP-selected lines was not prolonged.

Selecting *S. aureus* against melittin resulted in consistent patterns of mutations. All melittin-resistant strains had the following four mutations: stop gains for *pmt*R, missense for *bce*B, frameshift for *atl* and synonymous for *nam*A gene. Mutation in *pmt*R was identical to the stop-gain mutation described in melittin-selected *S. aureus* JLA513 (Dobson et al., 2013) and in tenecin-selected *S. aureus* SH1000 (Makarova et al., 2018). Bacteria harboring a mutation in the gene encoding GntR-type transcriptional repressor, PmtR (Joo et al., 2016b), can continuously efflux AMPs (Cheung and Otto, 2018; Cheung et al., 2018) along with Phenol-soluble modulins (PSMs), a bacterial secreted cytotoxin (Joo et al., 2016a). Mutation in *nam*A, a gene encoding flavin oxidoreductases, was previously reported for antibiotic stress response (Loi et al., 2019). All pexiganan-selected lines had mutations in *atl* and *men*F genes. Autolysis decreased in an *atl* (Bi functional autolysin gene) *S. aureus* mutant (Schlag et al., 2010). Some pexiganan-selected lines had a mutation in *vra*F (*bce*A) gene, *rpo*A gene or both. The *vra*FG gene (also designated as *bce*AB gene) codes for bacitracin export permease protein VraFG (BceAB), an ABC transporter controlling BceSR, a bacterial two-component systems (TCSs) associated with antimicrobial susceptibility (Yoshida et al., 2011). VraFG transporter sense the presence of cationic AMPs and transmit signaling through BceS (also known as NsaS and GraS) to activate BceR (also known as NsaR and GraR)-dependent transcription (Falord et al., 2012). Increased levels of phosphorylation due to point mutations in the *bce*SR (also known as *nsa*SR) operon leads to constitutive expression of BceAB/VraGF which facilitates detoxification by efflux (Coates-Brown et al., 2018). A mechanism by which *S. aureus* can evolve resistance to nisin (Arii et al., 2019), bacitracin (Hiron et al., 2011), and likely to other AMPs (Johnston et al., 2016; Makarova et al., 2018) such as human host defense peptides. Such resistance is a prerequisite for establishing chronic infection (Chaili et al., 2015). Many of the mutations identified herein were described previously in *S. aureus* clinical isolates from patients (Hafer et al., 2012). In summary, such mutations facilitate acquisition of drug resistance, contribute to immune evasion or alter host immune function (DeLeo et al., 2009). It is noteworthy that a recent large-scale whole-genome comparison in *Pseudomonas aeruginosa* showed that experimental antimicrobial resistance evolution reflects and predicts changes in naturally evolved clinical isolates (Wardell et al., 2019).

By analyzing the data with a four-parameter pharmacodynamic model (Regoes et al., 2004), we found that the Hill coefficient, kappa, evolves. Despite its potential importance (Yu et al., 2018; Firsov et al., 2003), kappa is often missed in many pharmacodynamic models, where it is set to 1 or another fixed value for all drugs and referred to as the sigmoidal constant (Craig, 1998; Thomas et al., 1998; Lenhard et al., 2015), even though kappa differs distinctly for different drugs (Yu et al., 2016). Using a different modeling approach, Chevereau et al. (2015) tested a dose-response curves of 78 genome-wide *Escherichia coli* gene deletion strains for a number of antibiotics. They found that the steepness of the dose-response curve varied with drug, but dose-sensitivity (the Hill coefficient *k* or *n* as denoted in Chevereau et al. (2015) of the mutants was the same as that of the wild types. Their strain collection did not contain strains which were specifically selected for resistance against the tested drugs. Moreover, in contrast to our approach, they modeled the pharmacodynamics for positive growth only and continuously adjusted the drug concentration. Pharmacodynamic parameters, including the Hill coefficient, play an important role in determining the population size of persistent *S. aureus* (Johnson and Levin, 2013) and *P. aeruginosa* (Hengzhuang et al., 2012) and therefore the probability of infection. In previous work it has been shown that the Hill coefficient relates to the probability of resistance evolution against AMPs (Yu et al., 2018). Whether or not a change in the Hill coefficient, driven by bacterial mutations, contributes to the speed and probability of resistance evolution will require additional work.

Kappa of pexiganan resistant strains, with mutations in *vra*F (*bce*A) and/or *rpo*A genes, were markedly high resulting in a very steep pharmacodynamic curve. Additionally, killing curves of melittin-selected strains, which showed cross resistance toward pexiganan, were as steep as pexiganan resistant strains. Interestingly, pexiganan-selected *S. aureus* which harbor mutations in *men*F and *atl* genes only, uniquely recorded extremely low kappa values. Linking variations in kappa values among AMP resistant strains and whether resistance mutations *per se* are costly or mitigated by compensatory mutations needs farther investigation. The steepness of the pharmacodynamic curve (kappa) determines the width of the mutation selection window MSW (Yu et al., 2018). Firsov et al. (2003, 2006) showed that the size of the (MSW) in *S. aureus* clinical isolates correlates positively with selection for resistance in fluoroquinolones, vancomycin, and daptomycin. We can conclude that MSW as inferred by kappa relies on both tested drug type and bacterial genetic background.

The current study is a proof of principle that shows that pharmacodynamics do evolve beyond MIC with AMP resistance. Applying this finding to all kind of host pathogen interactions and investigating co-evolution to host defense antimicrobial peptides or other antimicrobials will be useful to understand the dynamics of drug resistance evolution. For example, *S. aureus* on the skin are continuously under selection pressure from human AMPs. Along with *in vivo* infection dynamics studies (El Shazely et al., 2019), our study might shed some light on understanding host pathogen relationship during persistent infection. Understanding how antimicrobial peptides eradicate bacteria in physiological system, necessitates studying pharmacodynamics of AMPs *in vitro* (Yu et al., 2016) and *in vivo* (Zanchi et al., 2017).

## Data Availability Statement

The datasets generated for this study can be found in the NCBI – bioproject accession number is PRJNA399645.

## Author Contributions

BE, GY, and JR conceived the study. BE carried out the experiments and led the statistical analysis. PJ led the genomic sequencing. PJ and BE analyzed the genomic data. GY and BE carried out modeling of killing curves (MCMC predictions). BE and JR wrote the manuscript.

## Conflict of Interest

The authors declare that the research was conducted in the absence of any commercial or financial relationships that could be construed as a potential conflict of interest.

## Supplementary Material

FIGURE S1 Fitness costs inferred by growth parameters over mutation/operon in relation to the selective pressure treatment. [Vmax **(A)**, lag phase **(B)**, final OD **(C)**].

FIGURE S2 Time-kill curves of AMP selected *S. aureus* (SH1000) versus non-selected controls exposed to various concentrations of pexiganan. See Supplementary Table S1 for a full list of tested strains.

FIGURE S3 The pharmacodynamic curves of AMP resistant versus AMP sensitive *S. aureus* segregated by treatment **(A)** or by mutation **(B)**. The curves illustrate the effects (reflected as net bacteria growth rate) of increasing the concentration of pexiganan. The ribbon represents 95% of confidence interval.

TABLE S1 List of the MIC values of pexiganan and melittin resistant strains and their procedural controls tested against pexiganan and melittin. The MIC values of tenecin 1- and tenecin 1 + 2-evolved strains as well as their unselected controls tested against pexiganan only are listed below. The results were determined by a two-fold dilution protocol.

TABLE S2 Mutation/operon list of pexiganan and melittin resistant *S. aureus* strains and their procedural controls. (M: melittin-evolved strains, P: pexiganan- evolved strains, C: procedural control unselected strains, 1: present, 0: absent).
