# Supplementary figures and images for: Resistance Evolution Against Antimicrobial Peptides in Staphylococcus aureus Alters Pharmacodynamics Beyond the MIC
Source: Front Microbiol. 2020 Feb 14;11:103. doi: 10.3389/fmicb.2020.00103 (PMC7033599; doi:10.3389/fmicb.2020.00103)

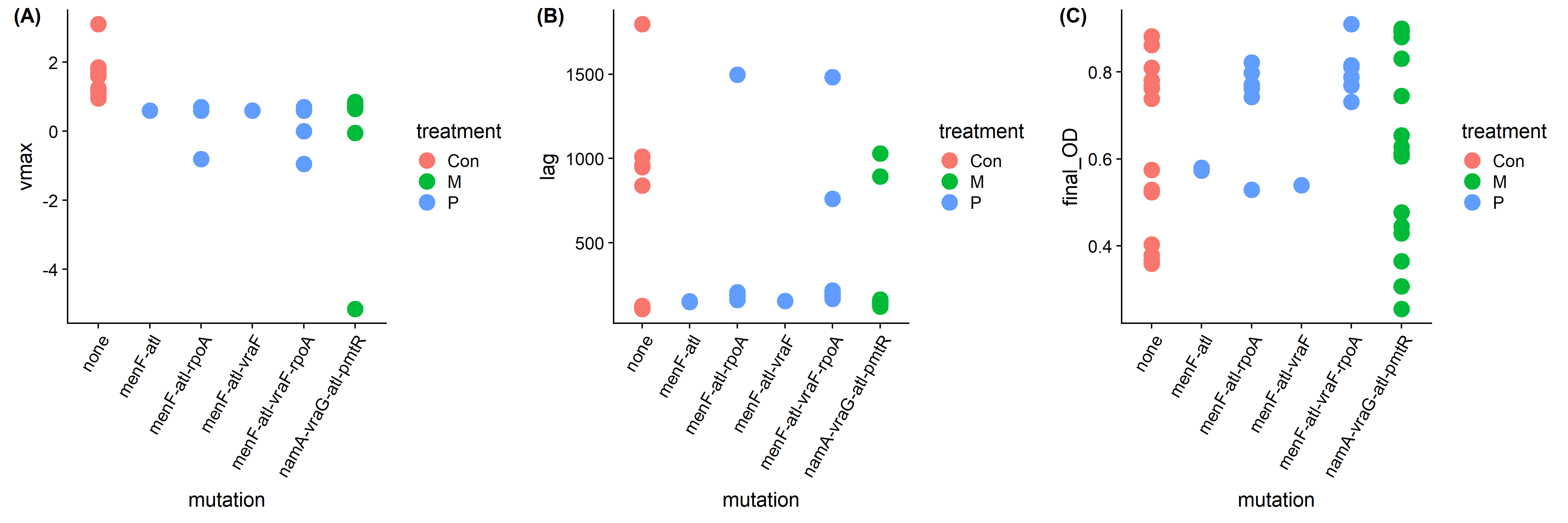

Supplement: FIGURE S1 — Fitness costs inferred by growth parameters over mutation/operon in relation to the selective pressure treatment. [Vmax (A), lag phase (B), final OD (C)]. [file Image_1.TIFF]

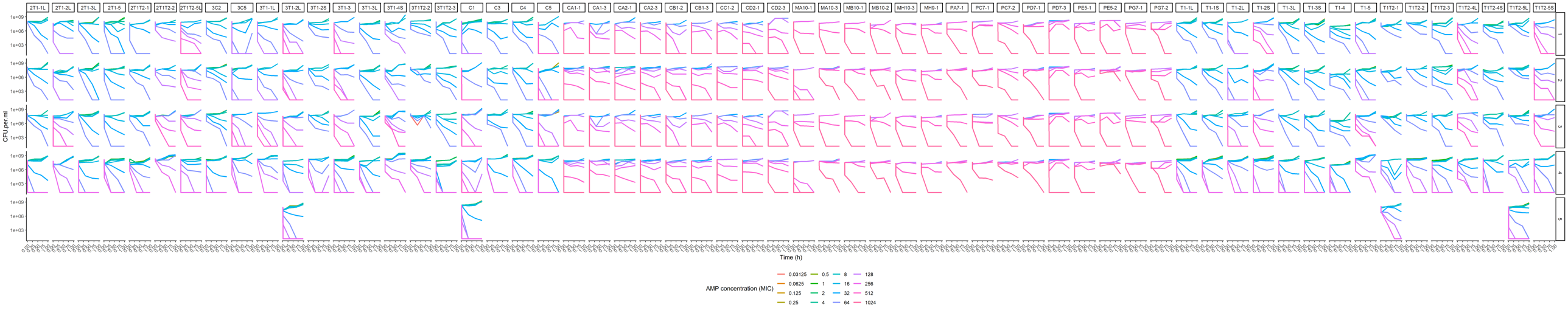

Supplement: FIGURE S2 — Time-kill curves of AMP selected S. aureus (SH1000) versus non-selected controls exposed to various concentrations of pexiganan. See Supplementary Table S1 for a full list of tested strains. [file Image_2.TIFF]

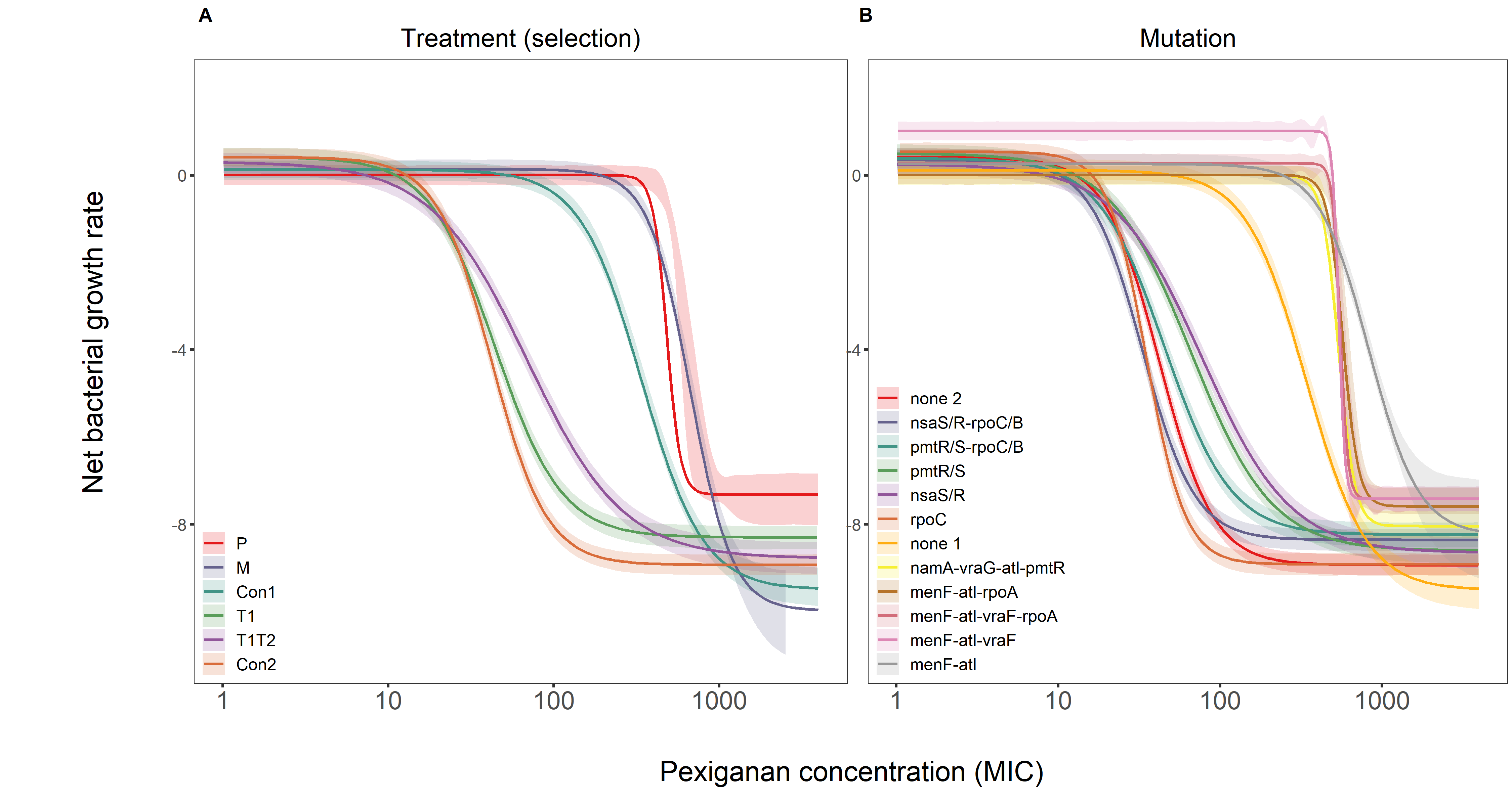

Supplement: FIGURE S3 — The pharmacodynamic curves of AMP resistant versus AMP sensitive S. aureus segregated by treatment (A) or by mutation (B). The curves illustrate the effects (reflected as net bacteria growth rate) of increasing the concentration of pexiganan. The ribbon represents 95% of confidence interval. [file Image_3.tiff]
